# Brucella epidydimo-orchitis successfully treated with dual oral drug regimen: A case report with differential diagnoses of malignancy and tuberculosis

**DOI:** 10.1016/j.radcr.2022.07.012

**Published:** 2022-07-27

**Authors:** Renad S. Nahas, Abdulsalam Alsulami, Manar O. Lashkar, Abrar K. Thabit

**Affiliations:** a Pharmacy Practice Department, Faculty of Pharmacy, King Abdulaziz University, 7027 Abdullah Alsulaiman St, Jeddah 22254-2265, Saudi Arabia; b Department of Pediatrics, Faculty of Medicine, King Abdulaziz University, 7027 Abdullah Alsulaiman St, Jeddah 22254-2265, Saudi Arabia

**Keywords:** Brucellosis, Brucella Epidydimo-orchitis, *Brucella orchitis*, Testis, Testicles

## Abstract

Brucellosis is a zoonotic disease caused by *Brucella* spp. When complicated, *Brucella* may affect any organ system, including the genitourinary system in the form of epidydimo-orchitis. *Brucella* orchitis is the second most common form of complicated brucellosis. The present case is for an adolescent who is otherwise healthy but presented with right testicular pain. Ultrasound imaging showed heterogeneous enlarged right testis with large heterogeneous mass and central necrosis. α-fetoprotein was normal and β-human choriogonadotropin was negative. Malignancy and tuberculosis were excluded based on histopathology and microbiology of the tissue biopsy, respectively. The history of raw dairy consumption and positive serology for *B. melitensis* and *B. abortus* established the diagnosis of *Brucella* epidydimo-orchitis. Treatment was successful with doxycycline and rifampin for four weeks. In pediatrics, it is important to rule out malignancy and make every attempt to avoid orchidectomy by making necessary investigations and involving infectious diseases consultation.

## Introduction

Brucellosis is a zoonotic disease commonly known as Malta fever. Brucellosis is an endemic disease in the Mediterranean region, Latin America, and the Middle East. Brucellosis is caused by Brucella pp., which are Gram-negative coccobacilli, non-sporing, and non-motile bacteria. There are four Brucella species known to cause disease in human, including *B. abortus, B. melitensis, B. suis*, and *B. canis* [1]. The most common way of brucellosis acquisition is through consumption of unpasteurized/raw dairy products and direct contact with infected livestock animals [2].

Brucellosis may manifest with fever, night sweats, headache, anorexia, and weight loss. When complicated, *Brucella* may affect any organ system, including osteoarticular, genitourinary, liver, and cardiovascular systems [3]. *Brucella* epidydimo-orchitis is the second most common form of complicated brucellosis, which can affect adults and pediatrics alike. Patients tend to present with urinary symptoms, scrotal pain, and swelling [4]. *Brucella* epidydimo-orchitis is treated for 6 weeks with 2 to 3 different antibiotics, which can include doxycycline, rifampin, trimethoprim/sulfamethoxazole, or ciprofloxacin [5, 6].

Only a few published cases of *Brucella* epidydimo-orchitis were identified in the literature. However, all the published cases were of adult patients. Therefore, this report describes an interesting successfully treated case of *Brucella* orchitis in an adolescent. This report was approved by the Biomedical Research Ethics Unit who also waived the requirement for patient consent.

## Case

**Fig. 1:**
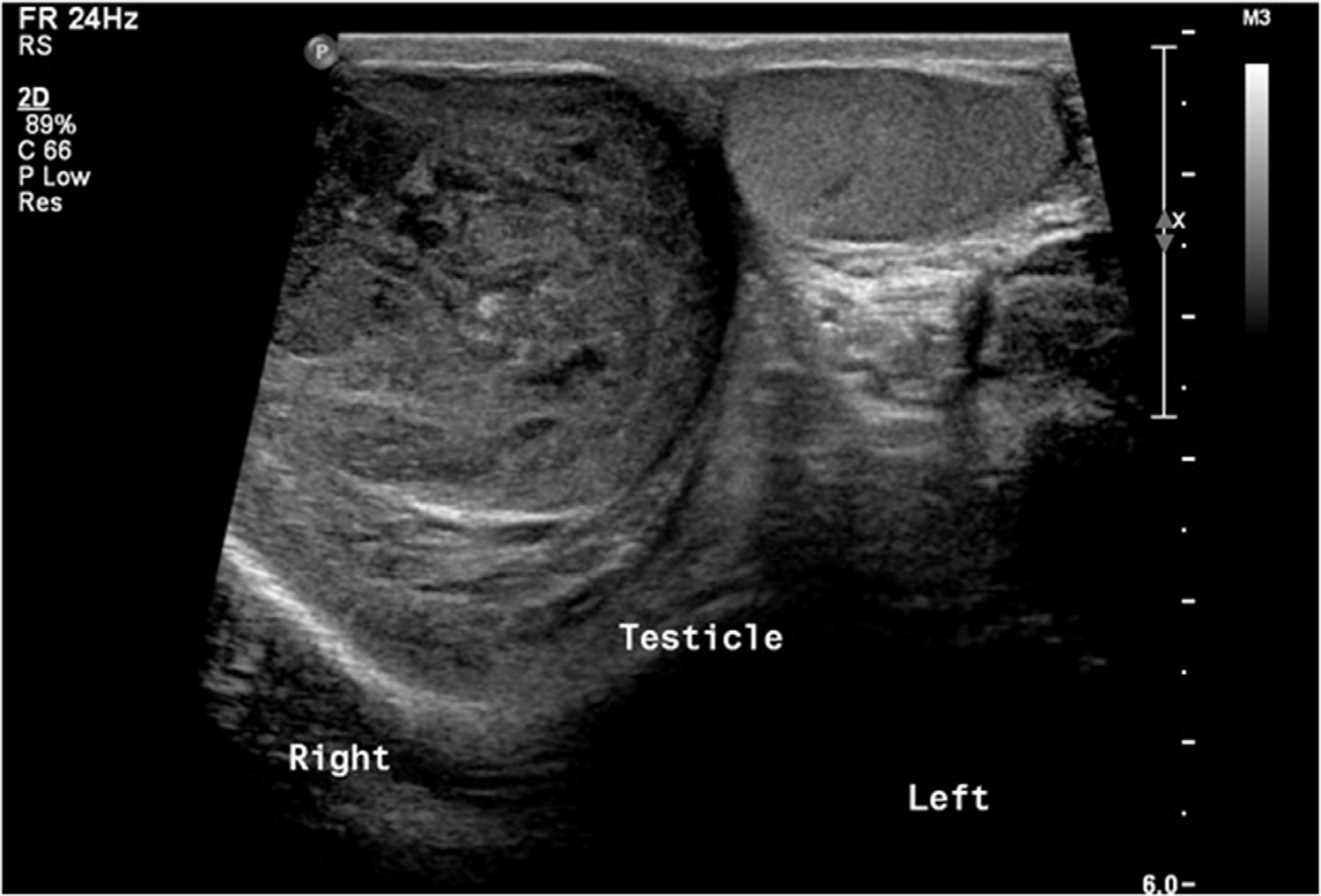
Grayscale ultrasound of the scrotum showed right testicular enlargement secondary to the mass.

**Fig. 2:**
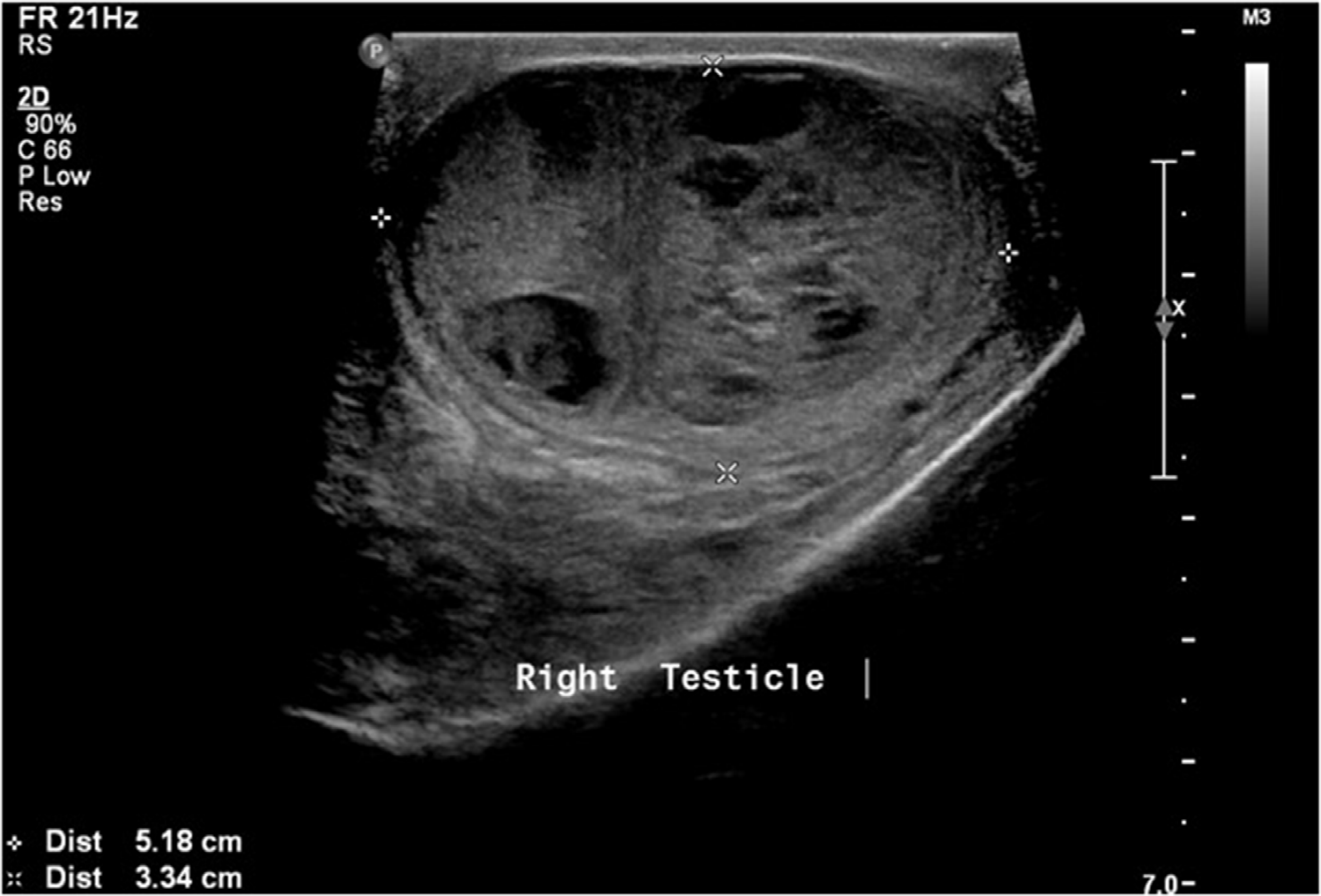
Grayscale ultrasound of the right testis in long axis showing the parenchymal heterogeneous mass lesion with areas of cystic changes/necrosis. Note the peripheral normal testicular tissue.

**Fig. 3:**
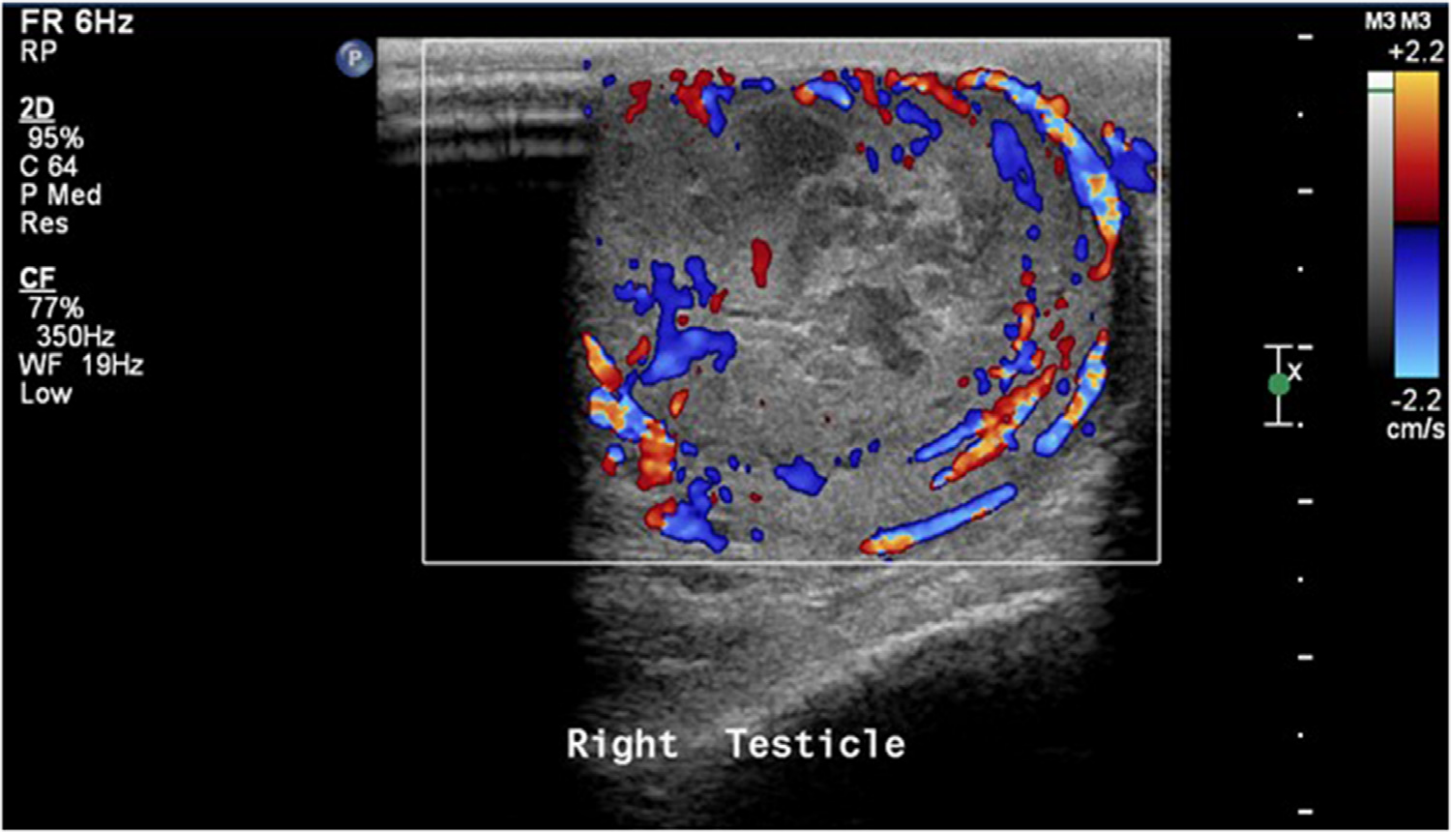
Doppler ultrasound of the same testis showing significant perilesional hyperemia denoting severe inflammation.

**Fig. 4:**
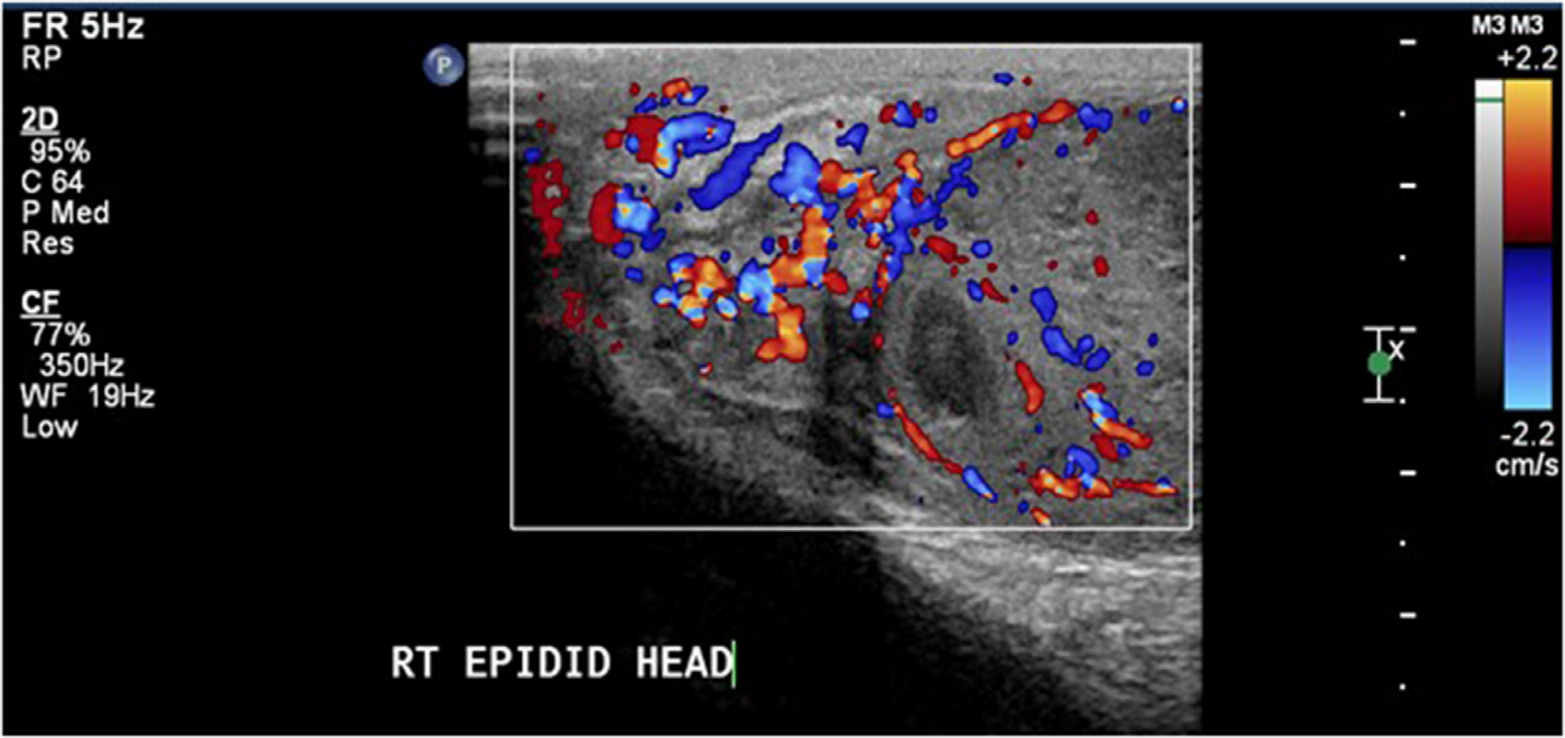
Doppler ultrasound image showing the severe hyperemia also involving the epididymis, in keeping with epidydimo-orchitis.

A 17-year-old male, previously healthy was seen in the outpatient clinic complaining of right testicular pain. He was afebrile and hemodynamically stable. Initial routine laboratory studies including complete blood count, urine analysis, urine culture, renal function, and electrolytes were normal. An ultrasound was obtained and showed a heterogeneous enlarged right testis measuring 5.22 × 3.4 × 3.9 cm with large heterogeneous mass and central necrosis measuring 4.6 × 2.7 cm (Fig. 1, Fig. 2, Fig. 3, Fig. 4). The right-sided venous plexus measured 0.28 cm at rest and Valsalva 0.39 cm. α-fetoprotein was normal at 1.35 (normal 0-5.8) and β-human choriogonadotropin was negative. A testicular biopsy was done under general anesthesia with plan for possible orchidectomy due to concerns for malignancy and tuberculosis after worsening of his symptoms. The frozen biopsy was negative for malignancy, so core biopsy was sent to pathology. Orchidectomy was not done. The biopsy revealed predominantly non-caseating granulomatous epidydimo-orchitis with focus of necrosis, and the biopsy was negative for malignancy. Acid fast bacilli smear and culture of the biopsied tissue showed no mycobacteria. Blood polymerase chain reaction testing for *Mycobacterium tuberculosis* complex was also negative. Histochemistry stain was also negative for fungi.

The patient was referred to the infectious disease clinic, and *Brucella* serology were ordered given history of raw milk and cheese ingestion 3 weeks prior to the initial presentation. Serum *Brucella* antibody titers were 1:640 for *B. melitensis* and 1:160 for *B. abortus*. The diagnosis of *Brucella* epidydimo-orchitis was made. Hence, the patient was started on doxycycline 100 mg orally every 12 hours and rifampin 600 mg orally once daily based on body weight of 40 kg. After four months of therapy, the patient's right testis reversed back to normal size and titers dropped to 1:320 for *B. melitensis* and 1:80 for *B. abortus.* Serology was repeated 14 months after initial presentation and showed antibody titers of 1:40 for both species. No adverse effect of therapy was reported.

## Discussion

Brucellosis remains a common zoonotic disease in the Middle East. It affects people of all ages with incidence of 10%-11% in endemic areas; however, 11.56% of cases occur in patients under 14 years of age [2]. Direct contact with infected animals, intake of raw dairy products, and inhalation of aerosols are all ways for infection to spread to people [8]. The most common genitourinary consequences of brucellosis in males are orchitis and epididymitis.

A case series by Fatani et al included a case of *Brucella* epidydimo-orchitis in a 44-year-old man who complained of scrotal pain and fever. The patient was successfully treated with ceftriaxone, doxycycline, and rifampin [9]. A study by Akıncı and colleague included 17 *Brucella* epidydimo-orchitis patients. Scrotal discomfort and edema were the most prevalent symptoms. Antibiotic regimens varied among patients where most patients (10/17) received rifampin plus doxycycline. Antibiotic treatment showed clinical improvement in 15 of 17 of the patients. In 2 cases, orchidectomy was indicated. Relapse occurred in only one patient after 3 months of antibiotic therapy [7]. In a retrospective study of 28 patients with *Brucella* epidydimo-orchitis, the most reported symptoms were fever, testicular pain, and swelling. All but 3 patients were successfully treated with rifampin, doxycycline, and streptomycin combinations. Orchidectomy was performed on 2 of the 3 treatment-resistant patients [10]. Navarro-Martinez et al included 59 cases of *Brucella* epidydimo-orchitis in a retrospective study with patients’ average age of 34 years. Of the included patients, most patients received a combination of doxycycline plus either streptomycin, netilmicin or gentamicin. Testicular abscess was drained in 2 patients, and orchidectomy was performed in 3 others [5].

Fentes et al reported a 37-year-old male with fever, scrotal pain and swelling who was diagnosed with *Brucella* epidydimo-orchitis based on blood culture and serologic test. The patient was successfully treated with doxycycline for 6 weeks and intramuscular streptomycin for 3 weeks [11]. Another case from Australia of an 18-year-old patient who presented with fever, dysuria, urethral discharge, and loss of appetite. *B. melitensis* was isolated from blood culture on the 4th day of hospitalization confirming the diagnosis of *Brucella* epidydimo-orchitis. During his initial presentation, the patient was given a single dose of azithromycin 1 g and gentamicin 320 mg. However, once the diagnosis of brucellosis was established, he was switched to a 7-day course of intravenous gentamicin and a 6-week course of doxycycline for 16 weeks. At the 6-week follow-up, the patient achieved a complete recovery [12].

In our report, the patient had complicated brucellosis and was successfully treated with dual therapy of doxycycline and rifampin. The recommended regimen by the World Health Organization (WHO) for acute uncomplicated brucellosis in adults and children older than 8 years is either an aminoglycoside for 7 days with doxycycline 100 mg orally twice daily for 6 weeks or doxycycline 100 mg orally twice daily plus rifampin 600-900 mg orally once daily for 6 weeks [3]. To date, there is no specific regimen recommended by WHO for epidydimo-orchitis; hence, the same regimen of uncomplicated brucellosis could be used for treatment [3].

## Conclusion

Epidydimo-orchitis is a rare brucellosis complication. Therefore, it is important to rule out malignancy and make every attempt to avoid orchidectomy by making necessary investigations and involving infectious diseases consultation. Unpasteurized/raw dairy products and direct contact with diseased livestock animals are the most prevalent ways to contract brucellosis. There is no specific recommendation for *Brucella* epidydimo-orchitis treatment by the WHO. Nonetheless, based on findings from this case and results of previously published cases, *Brucella* epidydimo-orchitis can be treated using the same regimens used for uncomplicated brucellosis.
